# Association between multimorbidity and informal long-term care use in China: a nationwide cohort study

**DOI:** 10.1186/s12877-023-04371-6

**Published:** 2023-10-30

**Authors:** Shu Chen, Yafei Si, Katja Hanewald, Bingqin Li, Chenkai Wu, Xiaolin Xu, Hazel Bateman

**Affiliations:** 1 Australian Research Council Centre of Excellence in Population Ageing Research (CEPAR), University of New South Wales, Sydney, NSW 2052 Australia; 2 School of Risk & Actuarial Studies, University of New South Wales, Sydney, Australia; 3 Social Policy Research Centre, University of New South Wales, Sydney, Australia; 4 Global Health Research Centre, Duke Kunshan University, Kunshan, China; 5 Department of Big Data in Health Science, School of Public Health, Zhejiang University, Hangzhou, China; 6 Centre of Clinical Big Data and Analytics, The Second Affiliated Hospital, Zhejiang University School of Medicine, Hangzhou, 310058 China; 7 School of Public Health, Faculty of Medicine, The University of Queensland, Brisbane, Australia

**Keywords:** Multimorbidity, Informal long-term care, Socio-economic disparities, Regional disparities, Economic burden

## Abstract

**Background:**

The impact of multimorbidity on long-term care (LTC) use is understudied, despite its well-documented negative effects on functional disabilities. The current study aims to assess the association between multimorbidity and informal LTC use in China. We also explored the socioeconomic and regional disparities.

**Methods:**

The study included 10,831 community-dwelling respondents aged 45 years and older from the China Health and Retirement Longitudinal Study in 2011, 2015, and 2018 for analysis. We used a two-part model with random effects to estimate the association between multimorbidity and informal LTC use. Heterogeneity of the association by socioeconomic position (education and income) and region was explored via a subgroup analysis. We further converted the change of informal LTC hours associated with multimorbidity into monetary value and calculated the 95% uncertainty interval (UI).

**Results:**

The reported prevalence of multimorbidity was 60·0% (95% CI: 58·9%, 61·2%) in 2018. We found multimorbidity was associated with an increased likelihood of receiving informal LTC (OR = 2·13; 95% CI: 1·97, 2·30) and more hours of informal LTC received (IRR = 1·20; 95% CI: 1·06, 1·37), ceteris paribus. Participants in the highest income quintile received more hours of informal LTC care (IRR = 1·62; 95% CI: 1·31, 1·99). The estimated monetary value of increased informal LTC hours among participants with multimorbidity was equivalent to 3·7% (95% UI: 2·2%, 5·4%) of China’s GDP in 2018.

**Conclusion:**

Our findings substantiate the threat of multimorbidity to LTC burden. It is imperative to strengthen LTC services provision, especially among older adults with multimorbidity and ensure equal access among those with lower income.

**Supplementary Information:**

The online version contains supplementary material available at 10.1186/s12877-023-04371-6.

## Introduction

Multimorbidity, the coexistence of two or more chronic conditions, has become a prevalent global issue [1]. Ageing and relative socioeconomic deprivations are two leading determinants of multimorbidity [2]. The prevalence of multimorbidity increases with age and reaches over 50% among people aged 65 years or older globally [3]. Evidence in developed countries shows that those with higher socioeconomic status have a lower prevalence of multimorbidity [3–5]. People with multimorbidity often have poorer health outcomes [6, 7], driving increased utilisation of health services and expenditures in developed [8, 9] and developing countries [10–12]. Consequently, multimorbidity has become a global concern, given the accelerated pace of population ageing globally.

People with multimorbidity have also been found to have poorer functional outcomes [13, 14]. Physiologically, people with multimorbidity have been found to have elevated levels of blood inflammatory markers, which indicate a heightened susceptibility to functional disabilities, frailty, and premature death [15–17]. In addition, there is strong evidence that some non-communicable diseases (NCDs), such as stroke [18], arthritis [19, 20], dementia [21], and diabetes [22], can directly lead to or predict negative functional outcomes. Such evidence implies a high demand for long-term care (LTC), i.e., help with daily activities, especially among older people with multimorbidity. Studies thus far have focused on assessing whether having selected medical conditions, such as dementia and stroke, is associated with increased care dependency or increased LTC expenditures [23, 24]. A collection of studies conducted in Germany [25], Japan [26], and Scotland [27] considered whether multimorbidity is associated with higher LTC dependency, receipt of formal social care, or total formal LTC expenditures. Though available evidence shows a positive correlation, it has focused on formal LTC services and expenditures recorded by insurance claims data in developed countries. Informal care has been largely ignored, though it usually accounts for a larger proportion of the care provided to older people, especially in countries whose LTC systems are not fully established.

China has the largest older adult population in the world, faces a high burden of NCDs, and substantial regional disparities in economic development and health outcomes. The number of older people aged 60 and above reached 264 million and accounted for 18·70% of the total population in 2020 [28]. With regard to China’s health profile, the burden of NCDs has increased dramatically over recent decades, and NCDs contributed to around 85% of the total disease burden in 2019 [29]. Studies have found that the prevalence of multimorbidity is 40–70% among older people in China [11, 30–32]. There are tiered regional differences in terms of economic development and health outcomes between the eastern, central, and western provinces in China. For example, the 2020 gross domestic product (GDP) per capita in Beijing (east region) was 2·6 times that of Anhui (central region), and 4·6 times that of Gansu (west region) [33]. In 2015, the average healthy life expectancy was estimated to be around 78 years among males in Beijing, Tianjin, and Shanghai (eastern region), but only 69 years in Qinghai, Tibet, and Yunnan (western region) [34].

The Chinese government has started to work on establishing a comprehensive LTC system for accessible, affordable, and high-quality LTC for older people and outlined a three-tiered LTC system in 2011 in the 12th Five-Year Plan for National Economic and Social Development [35]. However, the LTC system in China is still in its infancy, with insufficient financing and workforce to provide formal LTC services [36]. Therefore, older people with disabilities primarily rely on informal LTC, defined as LTC provided by informal caregivers such as family members and relatives free of charge, for assistance with daily living activities [37]. However, the burden of informal LTC could be partially transferred to the LTC system once it is fully established and functioning. Considering the heavy burdens of NCDs and rapid population ageing in China, it is imperative that we understand how multimorbidity could impact the burdens of informal LTC. However, little evidence is available on the relationship between multimorbidity and informal LTC use in China.

In this study, we aim to assess the association between multimorbidity and informal LTC use in China and explore the socioeconomic and regional disparities. We used 8-year nationally representative household survey data collected by the China Health and Retirement Longitudinal Study (CHARLS) for analysis. We presented the prevalence of multimorbidity, analysed the association between multimorbidity and informal LTC use, and estimated the corresponding monetary value of the change in informal LTC hours. The heterogeneity of the effect driven by socioeconomic position (education and income) and region (economic development) was further explored via sub-group analysis.

## Methods

### Data and study participants

We used longitudinal survey data collected by the CHARLS in Waves 1, 3, and 4, conducted in 2011, 2015, and 2018, respectively, for analysis. The survey follows the design of the Health and Retirement Survey (HRS) in the US [38]. The CHARLS sample is a nationally representative sample of the population aged over 45 years living in households in China. The first wave of the survey was conducted in 28 provinces, and 17,708 participants responded [39]. More about the CHARLS design has been published elsewhere [40].

A total of 10,831 participants were included for analysis in the current study. We included participants with no missing values in key variables of interest and no loss to follow-up in Waves 3 and 4. Observations for Wave 2 were not included, as data on informal LTC use were not available. We further excluded respondents with abnormal values in informal LTC use (Figure S1 for the flowchart of study participants selection and Table S1 for the basic characteristics of the excluded participants).

### Multimorbidity

Multimorbidity was defined as the coexistence of two or more chronic conditions. The CHARLS has measured 14 physical and mental chronic conditions, and we included all of them to measure multimorbidity in the current study. The 14 chronic conditions were hypertension, diabetes or high blood sugar, dyslipidaemia, heart disease, stroke, cancer, chronic lung disease, liver disease, digestive diseases, kidney disease, arthritis, asthma, psychiatric problems, and memory-related diseases.

### Informal long-term care use

Informal LTC refers to LTC provided by informal caregivers free of charge. Informal LTC use was measured as self-reported hours of help/services received from informal caregivers to assist with activities of daily living (ADLs) or instrumental activities of daily living (IADLs) in the past month. Informal caregivers include spouses, children, grandchildren, and relatives. The hours of help/services provided by all recorded informal caregivers on ADL and IADL assistance were aggregated to generate the total hours of help/services received.

### Covariates

We controlled for a set of covariates in the model to adjust for common socioeconomic determinants of health status and of receipt of informal LTC. Covariates included age, sex, marital status, socioeconomic position (education and income), region of residency, number of co-residents, urban or rural residence, and whether covered by public health insurance. Marital status was coded as a dichotomous variable: with a companion (married or partnered) or without a companion (separated, divorced, widowed, or never married).

We measured socioeconomic position by education and income, respectively. Each respondent’s highest education level obtained was coded using a scale with three tiers: (1) primary school or lower, (2) junior middle school, and (3) high school (vocational school) and above. To address the issue of underreported income, we used annual total household consumption per capita as a proxy and split this into quintiles to measure the relative socioeconomic deprivation by income.

We categorised the 28 provinces based on their GDP per capita in 2020 and ranked them in tertiles (see Appendix for specific provinces in the three groups) to measure regional disparities. The GDP per capita of the three regions was 7,404·6, 9,254·8, and 13,604·7 USD, respectively, in 2020 (exchange rate: 1 USD = 6·70 CNY).

### Statistical analysis

We calculated the reported prevalence of multimorbidity by wave and, for each wave, by sex, age group, functional status, region, and socioeconomic position. We also conducted Chi-squared tests to assess the statistical difference among subgroups. In addition, we calculated the reported prevalence of the five leading chronic conditions among participants with multimorbidity to better understand their disease profiles.

We used a panel data analysis approach to estimate the association between multimorbidity and informal LTC use. To account for the zero-mass problem for informal LTC use, we chose a two-part model with random effects for analysis [41, 42]. Specifically, a logit model was used to estimate the probability of informal LTC use (0/1) for the first part. Conditioning on the use of informal LTC (for the first part), a negative binomial model was adopted to estimate the effect of multimorbidity on the intensity of informal LTC use (hours) for the second part. We treated the dependent variable, the hours of informal LTC use, as a count variable as the underlying data is discrete data that takes on countable and distinct values, and the distribution is highly skewed. Typically, modelling a dependent count variable with excessive zeros employs either Poisson or negative binomial models. We selected a negative binomial model, considering the over-dispersion issue of informal LTC use. Standard errors were clustered at the individual level to handle serial correlation. The model also controlled for covariates and period fixed effects (see Appendix for the econometric model). To explore the heterogeneity of the association driven by socioeconomic and regional disparities, we also conducted subgroup analysis by socioeconomic position and region, respectively, using the same model. Considering the mediating role of functional disabilities in the relationship between multimorbidity and informal LTC use, we further assessed the association between multimorbidity and functional disabilities for a better interpretation of the results (Appendix).

We reported odds ratios (OR) for the first part’s logit model results and incidence rate ratios (IRR) for the second part’s negative binomial model results, with 95% CI and statistical significance level. The proportion of missingness for the covariates was low (< 2%), except in the case of household consumption (14·1%, 30·4%, and 16·6% at Waves 1, 3, and 4, respectively). We therefore used the multivariate imputation by chained equations (MICE) approach to impute the missing values of household consumption for regression estimate based on key socioeconomic covariates (education, gender, and age) and 20 sets of imputations [43, 44]. We reported weighted results for multimorbidity prevalence and non-weighted results for regression analysis [31].

### Sensitivity analysis

We conducted sensitivity analyses to check the robustness of the regression results. First, we used a Poisson model for the second part to re-estimate regression results. Second, we adjusted the measurement of multimorbidity to code it as the total number of chronic conditions. Third, we used non-medical household consumption per capita as a proxy for measuring income quintiles, considering total household consumption might be driven up by bad health. We performed the same imputation methods for non-medical household consumption to deal with missing data.

### Economic burden estimation

We converted the change of hours of informal LTC received among older people with multimorbidity to monetary value through benchmarking the national average salaries of urban workers in health and social services (Appendix for calculation details and Table S2 for base case data inputs). The burden was further converted to a percentage of annual GDP for an intuitive understanding of its magnitude relative to the economy.

We performed uncertainty analysis to assess the impact of variables of interest on the economic burden. We allowed the three variables of interest to vary within set ranges (Appendix for the range of variables). The three variables were multimorbidity prevalence, average hours of informal LTC use among middle-aged and older people with multimorbidity, and average annual salaries for workers in health and social services. For each variable, we performed 1,000 random draws following a normal distribution, with its mean set at the base case scenario level within the range of the variable. The 95% uncertainty interval (UI) of the economic burden as a percentage of annual GDP was calculated, defined in terms of the respective estimates obtained from the 2.5th and 97.5th percentile of the estimated economic burden.

## Results

For the 10,831 participants included in the analysis, the mean age was 64·1 in 2018. Females comprised 52·6% of the study sample, and 66·0% of the study sample received no education beyond the primary school level. Participants residing in rural areas comprised 64·6% of the sample. The percentages of participants residing in regions by tertile of economic development were 35·4% (most deprived), 33·4%, and 31·2% (most affluent), respectively. The percentages of participants with difficulties in ADLs and IADLs were 20·6% and 25·8%, respectively, in 2018 (Table 1).

In 2018, the reported prevalence of multimorbidity was 60·0% (95% CI: 58·9%, 61·2%) among study participants (Table 2). It was significantly higher among females, participants of older age, and those with disabilities (p < 0·001). Notably, the reported prevalence rose to 81% among those with disabilities in ADLs. Furthermore, we observed that the proportion was highest among participants at the lowest education level but highest among those in the top income quintile (p < 0·001). As for regional disparities, the reported prevalence was lowest in the most developed region but highest in the moderately developed region (p < 0·001). The top five contributors to multimorbidity were arthritis, hypertension, digestive diseases, dyslipidaemia, and heart diseases (Figure S2).

**Table 1:** Characteristics of the study sample (N = 10,831) in 2011, 2015, and 2018

Variables	Wave 1 (2011)	Wave 3 (2015)	Wave 4 (2018)
	**N (%) or Mean (SD)**	**N (%) or Mean (SD)**	**N (%) or Mean (SD)**
Age^*^	57·3 (9·1)	61·1 (9·1)	64·1 (9·1)
Female	5,695 (52·6%)	5,695 (52·6%)	5,695 (52·6%)
Marital status			
With companion (married or partnered)	9,775 (90·3%)	9,434 (87·1%)	9,051 (83·6%)
Education			
Primary school or lower	7,144 (66·0%)	7,144 (66·0%)	7,144 (66·0%)
Junior middle school	2,410 (22·3%)	2,410 (22·3%)	2,410 (22·3%)
High school and above	1,277 (11·8%)	1,277 (11·8%)	1,277 (11·8%)
Income quintile			
1st quintile (lowest)	2,154 (19·9%)	2,224 (20·5%)	2,194 (20·3%)
2nd quintile	2,168 (20·0%)	2,157 (19·9%)	2,172 (20·1%)
3rd quintile	2,185 (20·2%)	2,136 (19·7%)	2,153 (19·9%)
4th quintile	2,167 (20·0%)	2,187 (20·2%)	2,158 (19·9%)
5th quintile (highest)	2,156 (19·9%)	2,119 (19·6%)	2,151 (19·8%)
Total hh consumption per capita (CNY)^*^	7,173·1 (9410·1)	13,596·6 (21,687·1)	16,762·4 (26,391·9)
Non-medical hh consumption per capita (CNY)^*^	5,857·0 (10,426·5)	11,602·7 (21,324·9)	13,328·1(27,869·4)
Receiving any informal LTC	8·9%	16·1%	20·5%
Hours of informal LTC received/month	11·5 (75·7)	47·4 (321·8)	72·0 (466·1)
No. of co-residents	3·7 (1·8)	3·1 (1·3)	2·8 (1·5)
Covered by public health insurance^*^	10,090 (93·2%)	9,893 (91·3%)	10,449 (96·5%)
Having any difficulties in ADLs^*^	1,445 (13·4%)	2,060 (19·0%)	2,228 (20·6%)
Having any difficulties in IADLs^*^	1,923 (16·0%)	2,199 (20·3%)	2,795 (25·8%)

Data source: Harmonised CHARLS Data, 2011, 2015, 2018.

^*^These variables have missing values. The percentage of missing values for age, health insurance, and ADL/IADL difficulties was below 2%. A total of 14 observations were missing (0·1%) for age in the three waves; 35 (0·3%), 4 (< 0·1%), and 1 (< 0·1) observation(s) missing for health insurance coverage at Wave 1, 3, and 4, respectively; 113 (1·0%), 38 (0·4%), and 2 (< 0·1%) observations missing for having difficulties at ADLs at Wave 1, 3, and 4, respectively; 15 (0·1%) and 139 (1·3%) observations missing for having difficulties in IADLs at Wave 1 and 3, respectively. Percentages of missing observations for total household consumption were 14·1%, 30·4% and 16·6% at Wave 1, 3, and 4, respectively, while percentages for non-medical consumption were 19·6%, 37·6%, and 18·8%, respectively, in the three waves.

**Table 2:** Multimorbidity prevalence among people aged 45 years or older in China in 2011, 2015, and 2018

	2011		2015		2018	
	**Prevalence**	**p-value**	**Prevalence**	**p-value**	**Prevalence**	**p-value**
Total	35·2% (95% CI: 34·1%, 36·3%)		53·5% (95% CI: 52·4%, 54·7%)		60·0% (95% CI: 58·9%, 61·2%)	
Sex		< 0·000		< 0·000		< 0·000
Male	32·2%		50·7%		57·2%	
Female	38·5%		57·0%		62·5%	
Age group		< 0·000		< 0·000		< 0·000
<=50	26·4%		40·8%		45·7%	
50–59	35·4%		48·9%		51·6%	
60–69	43·2%		60·3%		63·4%	
>=70	42·8%		62·1%		68·2%	
Having difficulties in ADLs		< 0·000		< 0·000		< 0·000
Yes	54·9%		72·2%		81·0%	
No	32·3%		49·6%		54·6%	
Having difficulties in IADLs		< 0·000		< 0·000		< 0·000
Yes	51·2%		69·1%		78·0%	
No	32·1%		49·8%		53·8%	
Education		< 0·000		< 0·000		< 0·000
Primary school or lower	37·3%		56·1%		61·8%	
Junior middle school	31·5%		49·8%		56·7%	
High school and above	33·0%		50·0%		56·5%	
Income quintile		< 0·000		0·080		< 0·000
1 (lowest)	32·7%		52·7%		56·5%	
2	35·0%		53·8%		58·8%	
3	34·6%		52·5%		58·7%	
4	36·5%		56·3%		62·3%	
5 (highest)	38·9%		54·5		64·0%	
Region: economic development tertile		< 0·000		< 0·000		< 0·000
1st (most deprived)	36·4%		55·7%		61·1%	
2nd	39·9%		59·0%		65·2%	
3rd (most affluent)	29·8%		46·6%		53·5%	

Data source: Harmonised CHARLS Data, 2011, 2015, 2018.

We found multimorbidity to be significantly associated with a higher likelihood of receiving informal LTC (OR = 2·13; 95% CI: 1·97, 2·30) and receipt of more hours of informal LTC (IRR = 1·20; 95% CI: 1·06, 1·37), ceteris paribus. Participants with companions (married or partnered) and living in rural areas were significantly more likely to receive informal LTC, though they received fewer hours of care. Participants with higher levels of education and those who lived in the most affluent region were also found to be less likely to receive informal LTC (P < 0·001), and those in the most affluent region received significantly fewer hours of care (IRR = 0·78, 95% CI: 0·66, 0·91). However, those in the highest quintile of income received significantly more hours of informal LTC than those in the lowest quintile (IRR = 1·62; 95% CI: 1·31, 1·99). In addition, age and number of co-residents were both strong, significantly positive predictors in both parts of the model (Table 3).

**Table 3:** Regression results of the two-part model: multimorbidity and informal LTC use

Main model				
	**Probability of informal LTC use** (First part logit model)		**Intensity of informal LTC use** (Second part negative binomial model)	
Dependent variables	**OR**	**95% CI**	**IRR**	**95% CI**
Having multimorbidity	2·13***	(1·97, 2·30)	1·20**	(1·06, 1·37)
Female	1·57***	(1·44, 1·70)	0·95	(0·83, 1·09)
Age	1·06***	(1·05, 1·06)	1·02***	(1·02, 1·03)
Having companion	1·13**	(1·01, 1·26)	0·63***	(0·53, 0·75)
Number of co-residents	1·08***	(1·06, 1·11)	1·13***	(1·09, 1·18)
Education				
Ref (primary school or lower)				
Junior middle school	0·55***	(0·49, 0·62)	0·91	(0·76, 1·08)
High school and above	0·43***	(0·36, 0·51)	0·89	(0·65, 1·23)
Income quintile				
Ref (1st quintile, lowest)				
2nd quintile	1·02	(0·91, 1·14)	0·95	(0·78, 1·14)
3rd quintile	0·99	(0·89, 1·11)	1·23	(0·97, 1·57)
4th quintile	0·98	(0·88, 1·10)	1·12	(0·91, 1·39)
5th quintile (highest)	0·99	(0·88, 1·12)	1·62***	(1·31, 1·99)
Region: economic development tertile				
Ref (1st tertile, the most deprived)				
2nd tertile	0·93	(0·85, 1·02)	0·87**	(0·75, 1·00)
3rd tertile	0·71***	(0·64, 0·78)	0·78**	(0·66, 0·91)
Living in rural area	1·36***	(1·25, 1·49)	0·82**	(0·70, 0·96)
Covered by gov health insurance	0·83**	(0·72, 0·95)	0·90	(0·72, 1·14)

Data source: Harmonised CHARLS Data, 2011, 2015, 2018.

Note: *Statistically significant at 10%

**Statistically significant at 5%

***Statistically significant at 1%.

**Fig. 1:**
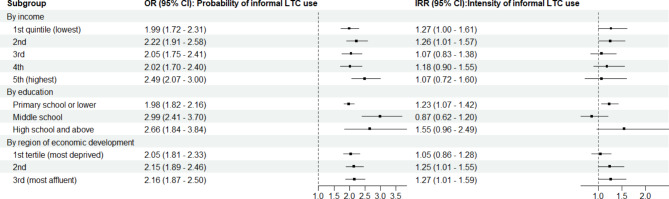
Subgroup analysis results of the two-part models by socioeconomic position and region. (Data source: Harmonised CHARLS Data, 2011, 2015, 2018)

**Fig. 2:**
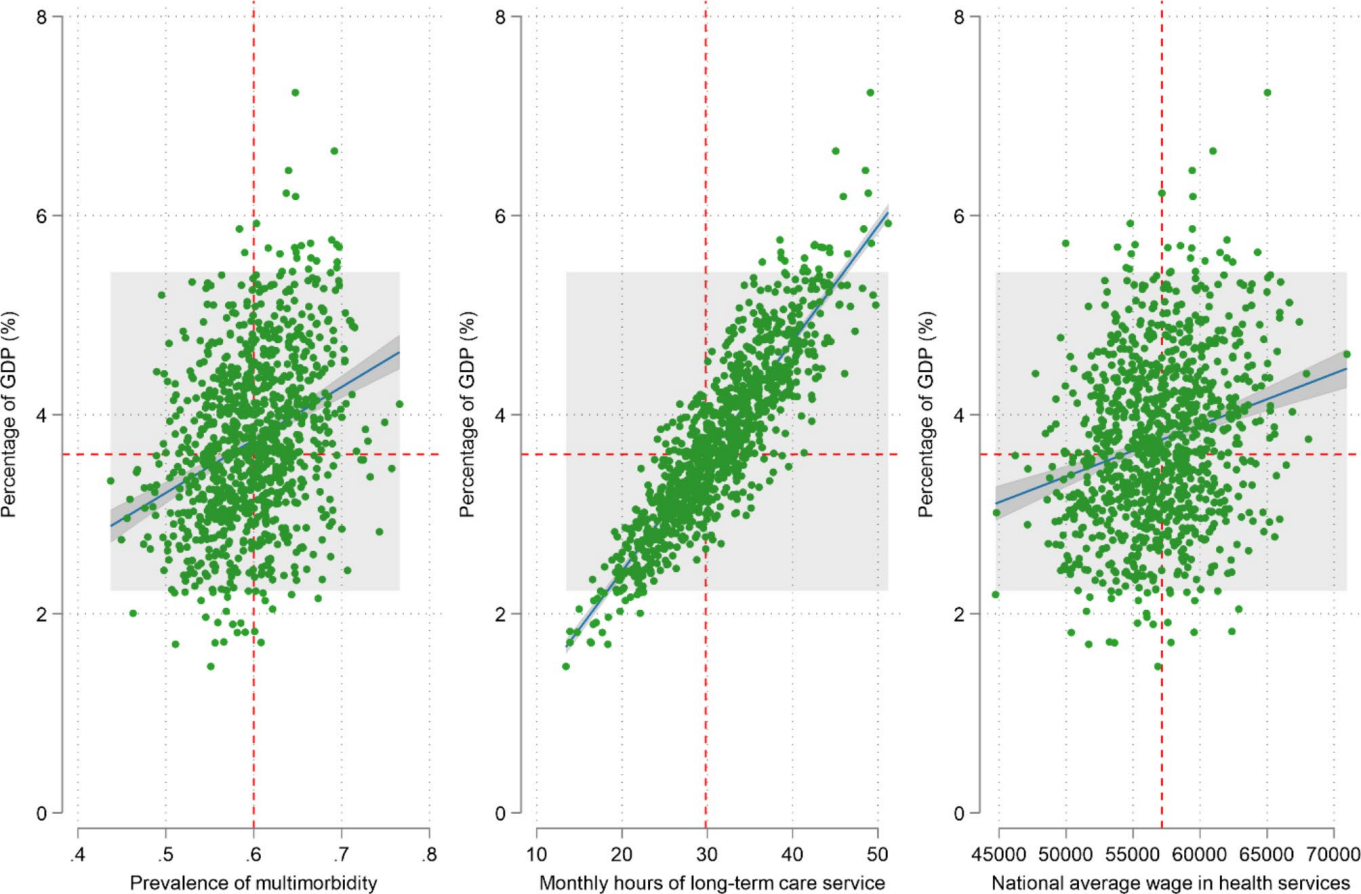
Uncertainty analysis results of the economic burden estimation, 2018. (Data source: Harmonised CHARLS Data, 2011, 2015, 2018. Note: The shaded area represents the 95% uncertainty interval)

Overall, the subgroup analysis detected no significant heterogeneity of effect by socioeconomic position or region (Fig. 1). Multimorbidity was associated with a higher likelihood of receiving informal LTC at all levels of socioeconomic groups and in all regions. Nevertheless, the effect on the intensity of informal LTC did not persist at the higher level of income groups. In addition, the results of the sensitivity analyses yielded highly consistent results (Table S4, S5 and S6). The estimated economic burden associated with increased informal LTC among middle-aged and older adults with multimorbidity was 512·2 billion USD in 2018, equivalent to 3·7% (95% UI: 2·2%, 5·4%) of China’s 2018 GDP (Fig. 2).

## Discussion

Despite the rich evidence on the impact of multimorbidity on health systems, this is, to our knowledge, the first study estimating the association between multimorbidity and informal LTC use in China. We find that the prevalence of multimorbidity was high and had risen fast among the study participants from 2011 to 2018. Overall, three of every five people aged 45 years or older in China reported multimorbidity. This is within the range of prevalence found in previous studies in China [11, 12, 30–32] but higher than the prevalence in developed countries [3]. Notably, the reported prevalence of multimorbidity rose to around 80% among participants with functional disabilities in 2018. The fast growth of reported multimorbidity prevalence can be attributed to worsened health conditions with ageing and improved diagnosis of NCDs due to strengthened NCDs control and management at the primary health care level. Strengthening the primary health care system is one focus of China’s health system reform, initiated in 2009; comprehensive control and management policies for multiple NCDs, strategies and projects have been implemented since then [45, 46]. Typical programs that facilitate NCD diagnosis include the nationwide Integrated NCD Prevention and Control Project and the take-up of free health checkups programs among older people [47, 48].

The results provide strong evidence that multimorbidity could increase the use of informal LTC, which in turn may have a profound impact on the LTC system, especially in terms of service delivery and financing when the LTC system begins to function well. Participants with multimorbidity are more likely to receive informal LTC and receive more hours of care. In addition, older participants and participants with a greater number of co-residents were significantly more likely to receive informal LTC and to receive more care. Those with a spouse or partner are more likely to receive informal LTC, though they receive fewer hours of informal LTC, as their functional abilities are better initially (Table S3). Notably, participants living in rural areas are also found to receive fewer hours of informal LTC, even though their functional abilities are worse than those of urban participants (Table S3). The annual economic burden due to increased informal LTC among people with multimorbidity is non-negligible, equivalent to 3·7% of China’s annual GDP in 2018. In comparison, total health expenditures, represented as a percentage of GDP, were 5·4% in 2018 [49]. This burden may be partially transferred to the LTC system, and it may make sustainable financing of the system even more challenging [36].

Socioeconomic position has been demonstrated to be an important determinant of health. Evidence shows it generally favours the less deprived in terms of access to and quality of health services utilised, as well as the health outcomes in Western countries [2, 4, 5, 50, 51]. Interestingly, our study finds that the socioeconomic disparities driven by income and education differ. First, the prevalence of multimorbidity increased with higher income but decreased with higher levels of education. One reasonable explanation is that people in the higher income groups are more likely to get diagnosed and to report it. In addition, our findings are consistent with those of previous studies conducted in China that the association between socioeconomic position and health could be mixed [11, 12]. The inverse association of multimorbidity by income and education could be driven by the difference in their interactions with unhealthy behaviours. For example, unhealthy behaviours such as smoking and high-frequency drinking were flat by wealth but are lower among older people with higher levels of education in China [52]. Second, participants in the highest income group received significantly more hours of informal LTC, though we found no significant difference in functional disabilities among participants in the different income groups (Table S3). Education, however, did not drive such differences in the hours of informal LTC received as income. This is consistent with previous studies showing that wealth is a stronger predictor of health services use and outcomes than education [51, 53].

We find regional disparities in terms of both the prevalence of multimorbidity and receipt of informal LTC. The reported prevalence of multimorbidity was lowest in the most developed region while highest in the moderately developed region, which consists primarily of central provinces. These findings are not surprising, as public health financing has been lowest for these provinces for years. In China, public health financing for provinces comes from a central government transfer fund, local government budgets, and a health insurance fund [45]. The most deprived provinces could receive the most central government transfers, while the most affluent provinces may utilise funding from their local budgets, leaving those provinces in the middle rank of economic development in the most disadvantageous positions in terms of health financing. For example, the 2018 total government health expenditures per capita in the three regions, from least to most developed, were 182·2, 163·3 and 207·5 USD (exchange rate: 1USD = 6·70 CNY, July 2022) [54]. The participants in the least affluent region were more likely to receive informal LTC and more hours of care, as participants in these provinces are found to have worse functional abilities compared to those in the most affluent region (Table S2).

This study’s findings carry important policy implications for chronic diseases and multimorbidity management in China. First and foremost, it is crucial to continue strengthening NCD prevention and control at the primary care level to reduce the prevalence of multimorbidity. Promoting healthy lifestyles is essential, and special attention should be paid to both the moderately and most deprived provinces, where the prevalence of multimorbidity is higher. Further, governments need to consider increasing health financing for these moderately developed provinces. Second, the management of NCDs should shift from targeting a single disease to multiple diseases, as has been widely pointed out [1, 2, 6, 11]. This is especially important in the case of older people, who are more prone to multimorbidity. Third, health and functional abilities are intercorrelated, and it is more efficient to integrate the provision of health and LTC services at the primary care level. For example, primary health centres should integrate timely and regular monitoring of functional abilities with health status for people with multimorbidity and take effective actions to restore or maintain these patients’ functional abilities. Fourth, resources should be purposively allocated to help the more socioeconomically deprived people, as measured by income, to receive health and LTC care in the important effort to reduce the socioeconomic inequalities which could be magnified in other aspects of people’s lives. The design of the public LTC insurance program should take this into consideration. Some of these implications may also apply to other developing countries with similar disease and demographic profiles.

The study admits to several limitations. First, we rely on the self-reported diagnoses by doctors from the CHARLS to measure multimorbidity, which might underestimate the multimorbidity prevalence as some participants, especially those in rural areas, might have limited access to timely diagnosis. Second, the numbers and types of chronic conditions measured in CHARLS are comprehensive though not exhaustive. Third, the measurement of multimorbidity focuses on the number of chronic conditions. Though standard and used extensively, it fails to capture the severity and interactions among different chronic conditions. Fourth, we excluded those lost to follow-up at Waves 3 and 4, which may lead to an underestimation of the results as they were generally older, had more difficulties in conducting ADLs and IADLs, and had a higher reported multimorbidity prevalence at baseline (Table S1). In addition, we only controlled marital status and the number of co-residents and were not able to include all the factors in our model that affect access to informal LTC, such as the willingness of family members to provide care, due to data availability. Further, the estimation of economic burdens is derived from the salaries of urban workers, which may overestimate the burden, despite our sensitivity analyses geared at partially offsetting this. The estimation also overlooks other sources of burden, such as the mental cost and the retirement savings foregone to caregivers, which may translate to other social burdens. Finally, future studies could explore the impact of multimorbidity on formal LTC burden when high-quality data become available.

## Conclusion

Our findings substantiate the threat of multimorbidity to LTC burden and highlight the socioeconomic inequalities in receiving informal LTC driven by income. Urgent actions should be taken, as this study proposes, to strengthen LTC services provision, especially among older people with multimorbidity and ensure equal access among those with lower income.

### Electronic supplementary material

Below is the link to the electronic supplementary material.

Supplementary Material 1

## Data Availability

The CHARLS data and socioeconomic data from China Statistical Yearbooks are all publicly available to researchers. Researchers can access and download the CHARLS data from its website (http://charls.pku.edu.cn/en/) and China’s socioeconomic data from the National Bureau of Statistics of China (http://www.stats.gov.cn/english/Statisticaldata/AnnualData/).
