# Supplementary material for: Association between multimorbidity and informal long-term care use in China: a nationwide cohort study
Source: BMC Geriatr. 2023 Oct 30;23:700. doi: 10.1186/s12877-023-04371-6 (PMC10617137; doi:10.1186/s12877-023-04371-6)
Supplement: Supplementary file 1 — Supplementary Material 1 [file 12877_2023_4371_MOESM1_ESM.docx]

**Association between multimorbidity and informal long-term care use**

**among older people in China: A nationwide cohort study**

Shu Chen MSc, Yafei Si MA, Katja Hanewald PhD, Bingqin Li PhD, Chenkai Wu PhD, Xiaolin Xu, PhD, Hazel Bateman PhD

**Appendix**

**Provinces in the three tertiles**

The first tertile (most deprived) contained 11 provinces, which were Gansu, Guangxi, Guizhou, Qinghai, Hebei, Henan, Heilongjiang, Jilin, Shanxi, Xinjiang, and Yunnan.

The second tertile had seven provinces, namely, Anhui, Hunan, Inner Mongolia, Jiangxi, Liaoning, Shaanxi, and Sichuan.

The third tertile (most developed) had ten provinces, which were Beijing, Chongqing, Fujian, Guangdong, Hubei, Jiangsu, Shandong, Shanghai, Tianjin, and Zhejiang.

**Our main econometric model**

The main econometric model is:

$$Y_{ijt}=\alpha+{\beta_{0}{mmorb}_{ijt}+\beta_{1}X}_{ijt}+\beta_{2}D_{t}+\varepsilon_{ijt}$$

Where $Y_{ijt}$is the outcome variable (number of informal LTC service hours received per month) for individual $i$ at community $j$ and time $t$, ${mmorb}_{ijt}$ is the variable of interest (0-absence of multimorbidity, 1-presence of multimorbidity; coded as the number of chronic conditions in sensitivity analysis), $X_{ijt}$ is the set of covariates, $D_{t}$the time dummies, and $\varepsilon_{ijt}$ is the error term that absorbs other unobservable shocks. Standard errors are clustered at the individual level for analysis.

**Measuring the association between multimorbidity and disabilities**

We assessed the association between having multimorbidity and disabilities to better understand the mechanism between multimorbidity and informal LTC use. We used a panel data analysis approach and an OLS regression model with random effects for analysis. The outcome variable was the number of ADLs or IADLs that the participants had difficulties with, and we ran two separate models to assess the relationship, respectively. The variable of interest was multimorbidity and was coded as a dichotomous variable. We controlled for the same set of covariates and the time-fixed effects as in the main model. Results are presented in Table S2.

**How to estimate the economic burden and set the range of the key variables for uncertainty analysis**

We calculated the total annual economic burden by multiplying the annual monetary value of the increased hours of informal LTC by the number of older people with multimorbidity. We set the range for the three key variables in the economic burden estimations using the principles as follows: 1) we relied on the 95% confidence interval of marginal effects of multimorbidity from the two-part fixed-effects regression models and set its range as 24.2-38.0 hours/per month; 2) based on a literature review on prevalence of multimorbidity among older people, we set the range to 45-75%; 3) we referred to the provincial difference of the annual salaries of urban workers in health and social services and set the range of salaries to 20% below and above the national average level (45,712-68,568 CNY/year).

**Table S1. Comparison of the basic characteristics of the included and excluded participants at baseline**

| Variables | Study sample    (N=10,831) | Excluded due to loss to follow up  (N=4,937) | Excluded due to missingness in outcome variable  (N=1,935) |
| --- | --- | --- | --- |
|  | **Mean (SD) or %** | **Mean (SD) or %** | **Mean (SD) or %** |
| Age | 57·3 (9·1) | 61·2 (11·9) | 59·2 (9·9) |
| Female (%) | 52·6% | 48.3% | 59.2% |
| Marital status (%) |  |  |  |
| With companion (married or partnered) | 90·3% | 81·7% | 84·7% |
| Education (%) |  |  |  |
| Primary school or lower | 66·0% | 63·7% | 76.4% |
| Junior middle school | 22·2% | 19.7% | 15.3% |
| High school and above | 11·8% | 16.6% | 8.4% |
| Multimorbidity prevalence (%) | 35·2% | 42.0% | 42.8% |
| Total hh consumption per capita (CNY) | 7,173·1 (9410·1) | 9,737.0 (35897.5) | 6396.8 (8658.1) |
| No. of co-residents | 3·7 (1·8) | 3·4 (1·8) | 3.6 (1·9) |
| Having any difficulties in ADLs | 13·4% | 20.9% | 23.1% |
| Having any difficulties in IADLs | 16·0% | 25.9% | 29.2% |

Data source: Harmonised CHARLS Data, 2011, 2015, 2018

**Table S2. Economic burden estimation: data inputs for the base case scenario, 2018**

| Key variables | Base case estimation inputs |
| --- | --- |
| Total population (billion) | 1·4 |
| Population aged 45 years or older | 41·4% |
| Multimorbidity (mbd) prevalence among people aged 45 years or older | 60·0% |
| Marginal increase of informal LTC use among mbd people (hours/month) | 31·1 |
| National average annual income in health and social services (CNY) | 57140·0 |
| Total annual GDP, China (billion, CNY) | 91928·1 |

Data source:

The data on population, population structure, salaries of urban workers in health and social services, and annual GDP were obtained from China Statistical Yearbook 2019. Estimates of informal LTC hours and multimorbidity prevalence were from the regression results of the current study

**Table S3. Regression models results: multimorbidity and having difficulties in ADLs and IADLs**

|  | **Coef.** | **P value** | **95% CI** | |  | **Coef.** | **P value** | **95% CI** | |
| --- | --- | --- | --- | --- | --- | --- | --- | --- | --- |
| **Dependent variable: ADL** |  |  |  |  | **Dependent variable: IADL** |  |  |  |  |
| Having multimorbidity | 0.20 | 0.000 | 0.18 | 0.22 |  | 0.20 | 0.000 | 0.18 | 0.22 |
| Female | 0.08 | 0.000 | 0.06 | 0.11 |  | 0.14 | 0.000 | 0.11 | 0.17 |
| Age | 0.02 | 0.000 | 0.01 | 0.02 |  | 0.02 | 0.000 | 0.02 | 0.02 |
| Having companion | -0.12 | 0.000 | -0.16 | -0.07 |  | -0.11 | 0.000 | -0.16 | -0.07 |
| Number of co-residents | 0.01 | 0.006 | 0.00 | 0.01 |  | 0.02 | 0.000 | 0.01 | 0.02 |
| Socioeconomic position: education |  |  |  |  |  |  |  |  |  |
| Ref (<lower secondary education) | - |  |  |  |  | - |  |  |  |
| Junior middle school | -0.11 | 0.000 | -0.14 | -0.08 |  | -0.16 | 0.000 | -0.19 | -0.13 |
| High school and above | -0.14 | 0.000 | -0.18 | -0.11 |  | -0.21 | 0.000 | -0.24 | -0.17 |
| Socioeconomic position: income |  |  |  |  |  |  |  |  |  |
| Ref (1^st^ quintile, lowest) | - |  |  |  |  | - |  |  |  |
| 2^nd^ quintile | 0.00 | 0.783 | -0.03 | 0.03 |  | 0.00 | 0.999 | -0.03 | 0.03 |
| 3^rd^ quintile | -0.02 | 0.227 | -0.05 | 0.01 |  | -0.01 | 0.693 | -0.04 | 0.03 |
| 4^th^ quintile | 0.00 | 0.829 | -0.03 | 0.03 |  | 0.01 | 0.578 | -0.02 | 0.04 |
| 5^th^ quintile (highest) | 0.00 | 0.784 | -0.03 | 0.03 |  | 0.02 | 0.307 | -0.02 | 0.05 |
| Geographic regions |  |  |  |  |  |  |  |  |  |
| Ref (1^st^, most deprived) | - |  |  |  |  | - |  |  |  |
| 2^nd^ | 0.02 | 0.149 | -0.01 | 0.06 |  | -0.02 | 0.238 | -0.05 | 0.01 |
| 3^rd^ (most affluent) | -0.08 | 0.000 | -0.11 | -0.05 |  | -0.10 | 0.000 | -0.13 | -0.07 |
| Living in rural area | 0.09 | 0.000 | 0.06 | 0.11 |  | 0.09 | 0.000 | 0.06 | 0.12 |
| Covered by gov health insurance | -0.04 | 0.051 | -0.07 | 0.00 |  | -0.01 | 0.534 | -0.05 | 0.03 |

Data source: Harmonised CHARLS Data, 2011, 2015, 2018

Note: we used OLS model to obtain the random-effects estimator.

**Table S4. Sensitivity analysis regression results: multimorbidity measured as number of chronic conditions**

| Main model | | | | |
| --- | --- | --- | --- | --- |
|  | **Probability of informal LTC use**  (First part logit model) | | **Intensity of informal LTC use**  (Second part negative binomial model) | |
| Dependent variables | **OR** | **95% CI** | **IRR** | **95% CI** |
| No. of chronic diseases | 1·29*** | (1·27, 1·32) | 1·06*** | (1·03, 1·09) |
| Female | 1·57*** | (1·44, 1·70) | 0·95 | (0·82, 1·09) |
| Age | 1·05*** | (1·05, 1·06) | 1·02*** | (1·02, 1·03) |
| Having companion | 1·14** | (1·02, 1·28) | 0·63*** | (0·53, 0·75) |
| Number of co-residents | 1·09*** | (1·06, 1·11) | 1·13*** | (1·09, 1·18) |
| Socioeconomic position: education |  |  |  |  |
| Ref (primary school or lower) | - | - | - | - |
| Junior middle school | 0·56*** | (0·49, 0·63) | 0·91 | (0·77, 1·09) |
| High school and above | 0·42*** | (0·35, 0·51) | 0·90 | (0·65, 1·24) |
| Socioeconomic position: income |  |  |  |  |
| Ref (1^st^ quintile, lowest) | - | - | - | - |
| 2^nd^ quintile | 1·01 | (0·90, 1·13) | 0·95 | (0·78, 1·14) |
| 3^rd^ quintile | 0·97 | (0·87, 1·09) | 1·21 | (0·95, 1·53) |
| 4^th^ quintile | 0·96 | (0·86, 1·08) | 1·10 | (0·89, 1·36) |
| 5^th^ quintile (highest) | 0·95 | (0·84, 1·08) | 1·57*** | (1·27,1·95) |
| Region: economic development |  |  |  |  |
| Ref (1^st^ tertile, the most deprived) |  |  |  |  |
| 2^nd^ tertile | 0·92 | (0·84, 1·01) | 0·86** | (0·74, 0·99) |
| 3^rd^ tertile | 0·73*** | (0·66, 0·81) | 0·78** | (0·66, 0·91) |
| Living in rural area | 1·39*** | (1·27, 1·52) | 0·83** | (0·71, 0·97) |
| Covered by gov health insurance | 0·82** | (0·71, 0·93) | 0·91 | (0·72, 1·16) |

Data source: Harmonised CHARLS Data, 2011, 2015, 2018

Note: *Statistically significant at 10%.

**Statistically significant at 5%.

***Statistically significant at 1%

**Table S5. Sensitivity analysis: two-part model results with the second part as Poisson model**

| Main model | First part logit model | | | |  | Second part truncated Poisson model | | | |
| --- | --- | --- | --- | --- | --- | --- | --- | --- | --- |
|  | **OR** | **P value** | **95% CI** | |  | **IRR** | **P value** | **95% CI** | |
| Having multimorbidity | 2.13 | 0.000 | 1.97 | 2.30 |  | 1.32 | 0.000 | 1.14 | 1.52 |
| Female | 1.57 | 0.000 | 1.44 | 1.70 |  | 0.98 | 0.823 | 0.82 | 1.17 |
| Age | 1.06 | 0.000 | 1.05 | 1.06 |  | 1.03 | 0.000 | 1.02 | 1.04 |
| Having companion | 1.13 | 0.027 | 1.01 | 1.26 |  | 0.67 | 0.001 | 0.54 | 0.84 |
| Number of person(s) living in the same household | 1.08 | 0.000 | 1.06 | 1.11 |  | 1.13 | 0.000 | 1.08 | 1.17 |
| Socioeconomic position: education |  |  |  |  |  |  |  |  |  |
| Ref (<lower secondary education) | - |  |  |  |  | - |  |  |  |
| Junior middle school | 0.55 | 0.000 | 0.49 | 0.62 |  | 0.88 | 0.234 | 0.71 | 1.09 |
| High school and above | 0.43 | 0.000 | 0.36 | 0.51 |  | 0.94 | 0.720 | 0.67 | 1.33 |
| Socioeconomic position: income |  |  |  |  |  |  |  |  |  |
| Ref (1^st^ quintile, lowest) | - |  |  |  |  |  |  |  |  |
| 2^nd^ quintile | 1.02 | 0.782 | 0.91 | 1.14 |  | 0.96 | 0.722 | 0.75 | 1.22 |
| 3^rd^ quintile | 0.99 | 0.873 | 0.89 | 1.11 |  | 1.13 | 0.422 | 0.83 | 1.55 |
| 4^th^ quintile | 0.98 | 0.763 | 0.88 | 1.10 |  | 1.04 | 0.736 | 0.81 | 1.34 |
| 5^th^ quintile (highest) | 0.99 | 0.910 | 0.88 | 1.12 |  | 1.52 | 0.001 | 1.19 | 1.94 |
| Geographic regions |  |  |  |  |  |  |  |  |  |
| Ref (1^st^, most deprived) | - |  |  |  |  | - |  |  |  |
| 2^nd^ | 0.93 | 0.120 | 0.85 | 1.02 |  | 0.95 | 0.534 | 0.81 | 1.12 |
| 3^rd^ (most affluent) | 0.71 | 0.000 | 0.64 | 0.78 |  | 0.81 | 0.061 | 0.65 | 1.01 |
| Living in rural area | 1.36 | 0.000 | 1.25 | 1.49 |  | 0.83 | 0.038 | 0.70 | 0.99 |
| Covered by gov health insurance | 0.83 | 0.006 | 0.72 | 0.95 |  | 0.92 | 0.554 | 0.70 | 1.21 |

Data source: Harmonised CHARLS Data, 2011, 2015, 2018

**Table S6. Sensitivity analysis regression model results: non-medical household consumption as proxy to measure income quintiles**

| Main model | First part logit model | | | |  | Second part truncated Poisson model | | | |
| --- | --- | --- | --- | --- | --- | --- | --- | --- | --- |
|  | **OR** | **P value** | **95% CI** | |  | **IRR** | **P value** | **95% CI** | |
| Having multimorbidity | 2.13 | 0.000 | 1.97 | 2.30 |  | 1.21 | 0.004 | 1.06 | 1.37 |
| Female | 1.57 | 0.000 | 1.44 | 1.70 |  | 0.97 | 0.631 | 0.84 | 1.11 |
| Age | 1.06 | 0.000 | 1.05 | 1.06 |  | 1.02 | 0.000 | 1.02 | 1.03 |
| Having companion | 1.13 | 0.027 | 1.01 | 1.26 |  | 0.64 | 0.000 | 0.53 | 0.76 |
| Number of person(s) living in the same household | 1.08 | 0.000 | 1.06 | 1.11 |  | 1.13 | 0.000 | 1.09 | 1.17 |
| Socioeconomic position: education |  |  |  |  |  |  |  |  |  |
| Ref (<lower secondary education) | - |  |  |  |  | - |  |  |  |
| Junior middle school | 0.55 | 0.000 | 0.49 | 0.62 |  | 0.91 | 0.282 | 0.76 | 1.08 |
| High school and above | 0.43 | 0.000 | 0.36 | 0.51 |  | 0.90 | 0.499 | 0.66 | 1.23 |
| Socioeconomic position: income |  |  |  |  |  |  |  |  |  |
| Ref (1^st^ quintile, lowest) | - |  |  |  |  | - |  |  |  |
| 2^nd^ quintile | 1.03 | 0.592 | 0.92 | 1.15 |  | 1.00 | 0.984 | 0.84 | 1.20 |
| 3^rd^ quintile | 1.01 | 0.875 | 0.90 | 1.13 |  | 1.07 | 0.603 | 0.84 | 1.35 |
| 4^th^ quintile | 0.96 | 0.480 | 0.86 | 1.08 |  | 1.17 | 0.130 | 0.95 | 1.44 |
| 5^th^ quintile (highest) | 1.00 | 0.993 | 0.88 | 1.13 |  | 1.55 | 0.000 | 1.26 | 1.91 |
| Geographic regions |  |  |  |  |  |  |  |  |  |
| Ref (1^st^, most deprived) | - |  |  |  |  | - |  |  |  |
| 2^nd^ | 0.93 | 0.120 | 0.85 | 1.02 |  | 0.88 | 0.069 | 0.76 | 1.01 |
| 3^rd^ (most affluent) | 0.71 | 0.000 | 0.64 | 0.78 |  | 0.79 | 0.005 | 0.66 | 0.93 |
| Living in rural area | 1.36 | 0.000 | 1.25 | 1.49 |  | 0.82 | 0.016 | 0.69 | 0.96 |
| Covered by gov health insurance | 0.83 | 0.006 | 0.72 | 0.95 |  | 0.92 | 0.479 | 0.73 | 1.16 |

Data source: Harmonised CHARLS Data, 2011, 2015, 2018

**Figure S1. Flowchart of the study sample selection**

17,708 study participants responded at baseline

5 study participants were excluded due to abnormal values^*^ in informal long-term use

4,937 study participants were excluded due to non-responsiveness at Wave 1, 3, or 4

1,935 study participants were excluded due to missing key variables of interest

10,831 participants were included in the final study sample

10,836 participants with no missing values in multimorbidity and informal long-term care use were included

12,771 study participants responded at Waves 1, 3, and 4

Note:

^*^Abnormal values are defined as those who received over 10,000 hours per month without suffering from any severe disabilities, i.e., having difficulties in two or fewer activities of daily living (ADLs) or instrumental activities of daily living (IADLs).

**Figure S2. Reported prevalence of top five diseases among participants with multimorbidity in 2011, 2015, and 2018**

Data source: Harmonised CHARLS Data, 2011, 2015, 2018
